# Mitochondrial-regulated Tregs: potential therapeutic targets for autoimmune diseases of the central nervous system

**DOI:** 10.3389/fimmu.2023.1301074

**Published:** 2023-12-12

**Authors:** Aoya Han, Tingting Peng, Yinyin Xie, Wanwan Zhang, Wenlin Sun, Yi Xie, Yunqing Ma, Cui Wang, Nanchang Xie

**Affiliations:** ^1^ Department of Neurology, The First Affiliated Hospital of Zhengzhou University, Zhengzhou, China; ^2^ Department of Clinical Laboratory, The First Affiliated Hospital of Zhengzhou University, Zhengzhou, China

**Keywords:** mitochondria, regulatory T cell, Foxp3, self-tolerance, central nervous system, autoimmune diseases

## Abstract

Regulatory T cells (Tregs) can eliminate autoreactive lymphocytes, induce self-tolerance, and suppress the inflammatory response. Mitochondria, as the energy factories of cells, are essential for regulating the survival, differentiation, and function of Tregs. Studies have shown that patients with autoimmune diseases of the central nervous system, such as multiple sclerosis, neuromyelitis optica spectrum disorder, and autoimmune encephalitis, have aberrant Tregs and mitochondrial damage. However, the role of mitochondrial-regulated Tregs in autoimmune diseases of the central nervous system remains inconclusive. Therefore, this study reviews the mitochondrial regulation of Tregs in autoimmune diseases of the central nervous system and investigates the possible mitochondrial therapeutic targets.

## 1 Introduction

Regulatory T cells (Tregs) are negative immune-regulatory cells that play a significant role in immune tolerance and the normal function of the immune system by eliminating the autoreactive lymphocytes, inducing self-tolerance, and suppressing the inflammatory response through various mechanisms (1, 2). Recent studies have also indicated the ability of Tregs to promote tissue repair or regeneration by secreting tissue-specific regenerative factors (3–5). Consistently, aberrant Tregs are a major driver of many autoimmune diseases. Reduced number and impaired function of Tregs have been reported in various autoimmune diseases, including myasthenia gravis, systemic lupus erythematosus, rheumatoid arthritis, and type 1 diabetes (6–8). Some autoimmune diseases of the central nervous system (CNS) occur due to self-tolerance defects. Self-tolerance defect in multiple sclerosis (MS) occurs mainly due to impaired Treg function, but there are also cases of decreased Treg number (6, 9, 10). However, neuromyelitis optica spectrum disorder (NMOSD) and autoimmune encephalitis are characterized by reduced number of Tregs (11, 12). The absence of immunosuppressive capacity of Tregs and decreased number of Tregs activate autoreactive cells, promote B cells to produce autoantibodies and effector T cells to secrete pro-inflammatory cytokines and chemokines, and induce the infiltration of macrophages and effector T cells into the CNS, ultimately promoting the development of autoimmune diseases of the CNS (6, 11–14). However, the specific mechanisms leading to reduced number and impaired function of Tregs in autoimmune diseases of the CNS remain to be determined.

Among numerous cellular biological processes and molecular mechanisms associated with Tregs, their unique metabolic profile has recently drawn significant interest. In physiological conditions, Tregs exhibit increased mitochondrial metabolism, characterized by high levels of mitochondrial fatty acid oxidation (FAO) and oxidative phosphorylation (OXPHOS) and modest glycolysis (15–19). During FAO and OXPHOS, FoxP3, a critical transcription factor of Tregs, is transcriptionally upregulated, which is essential for maintaining the immunosuppressive function and stability of Tregs and can promote Treg differentiation by inhibiting RORγt binding to DNA (15, 16, 20–23). Glycolysis is necessary for the growth and proliferation of Tregs, but it reduces the immunosuppressive ability and stability of Tregs during growth and proliferation (24–26). Glycolysis is also a key energy source for Treg migration to inflammatory tissue (27). Moreover, mitochondrial metabolite α-ketoglutarate is a substrate for ten-eleven translocase (TET)-mediated demethylation of the *FoxP3* locus in Tregs, which is required for optimal expression of FoxP3 and immunosuppressive function of Tregs (28–30). Mitochondrial damage, such as damaged respiratory chain complexes and abnormal mitochondrial morphology, can markedly impair the survival, differentiation, and function of Tregs. Therefore, maintaining mitochondrial structure and function is critical for Treg homeostasis and function.

Recent studies have found abnormal mitochondrial morphology, impaired cristae organization, reduced activity and expression of respiratory chain complexes, decreased expression of cytochrome C, increased content of mitochondrial DNA (mtDNA) and mitochondrial reactive oxygen species (mtROS), and damaged mitophagy in Tregs of patients with autoimmune diseases of the CNS such as MS and NMOSD (31–33). Therefore, mitochondrial-regulated Tregs may be involved in the occurrence and progression of autoimmune diseases of the CNS (31). This paper reviews the Treg regulation by mitochondria in autoimmune diseases of the CNS and introduces the possible mitochondrial therapeutic targets.

## 2 Mitochondrial regulation of Tregs in autoimmune diseases of the CNS

### 2.1 Accumulation of mtROS decreases the number of Tregs

**Figure 1:**
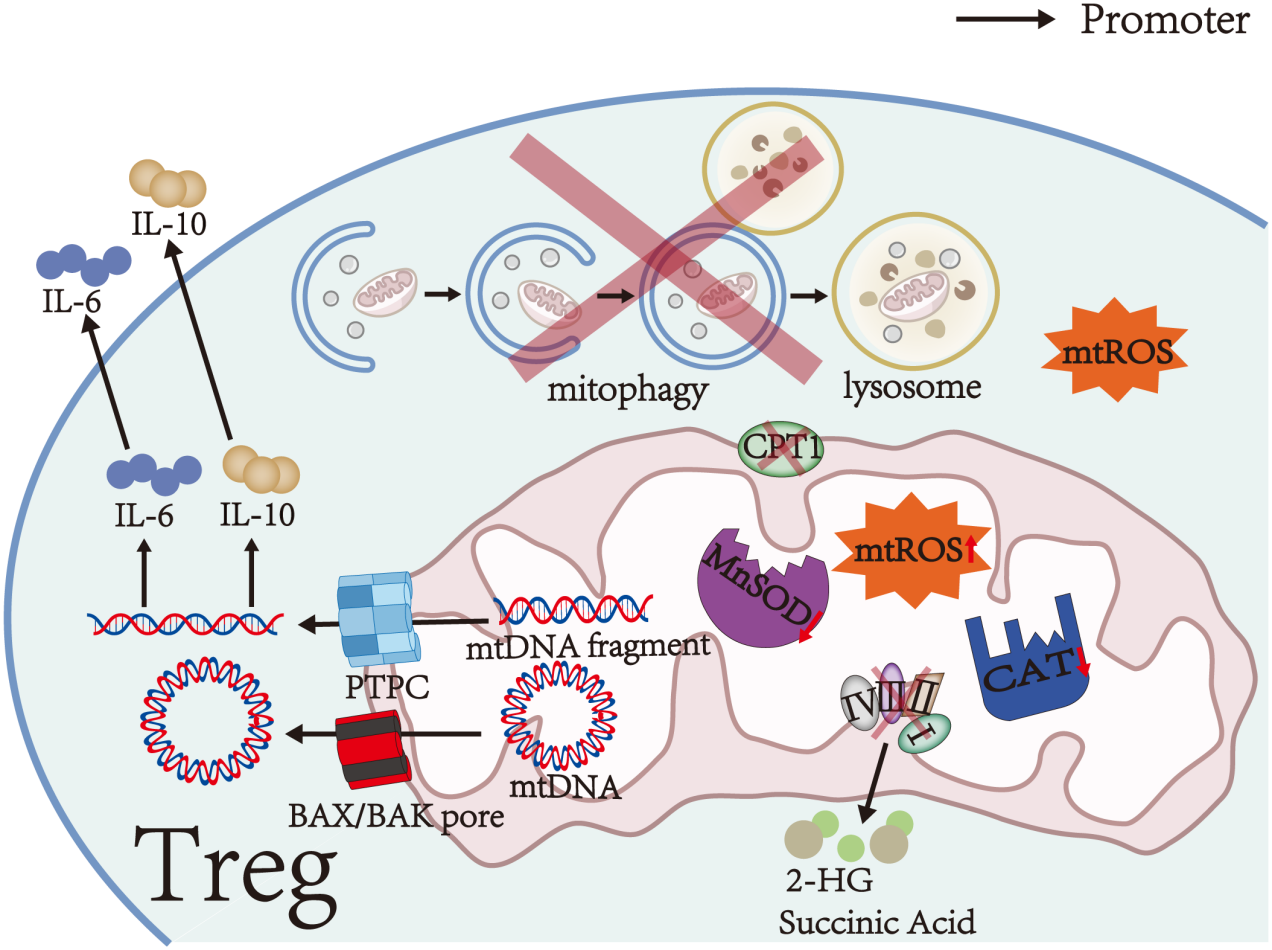
Mitochondrial damage in Tregs. Aberrant mitochondrial morphology, impaired cristae organization, reduced expression of respiratory chain complexes, and significantly decreased activities of MnSOD and CAT in Tregs cause mitochondrial dysfunction and mtROS accumulation. Furthermore, respiratory chain complex III deficiency leads to the accumulation of succinate and 2-HG in Tregs. During mitochondrial dysfunction, mtDNA and mtDNA fragments are released into the cytosol via the PTPC or BAX/BAK pores. Cytosolic mtDNA and mtDNA fragments can promote the secretion of IL-6 and IL-10. CPT1 on the outer mitochondrial membrane is a rate-limiting enzyme in FAO, which is the main energy source of Tregs. Therefore, reduced or defective CPT1 activity causes FAO impairment. Moreover, impaired initiation of mitophagy, incorrect autophagosome formation, or aberrant lysosomal degradation impairs mitophagy and leads to damaged mitochondria accumulation in Tregs.

As a signaling molecule, mtROS plays a significant role in activating signaling pathways and determining cell fate. mtROS level is strictly regulated by antioxidants such as superoxide dismutase and catalase (CAT) (34–36). However, the activities of manganese superoxide dismutase (MnSOD) and CAT are significantly decreased in Tregs of mice with experimental autoimmune encephalomyelitis (EAE), which impairs the ability of the antioxidant system to scavenge mtROS and results in mtROS accumulation (**Figure 1**) (31). Furthermore, aberrant mitochondrial morphology, impaired cristae organization, and reduced expression of respiratory chain complexes in MS and NMOSD can cause mitochondrial dysfunction and mtROS accumulation (**Figure 1**) (31–33, 37).

**Figure 2:**
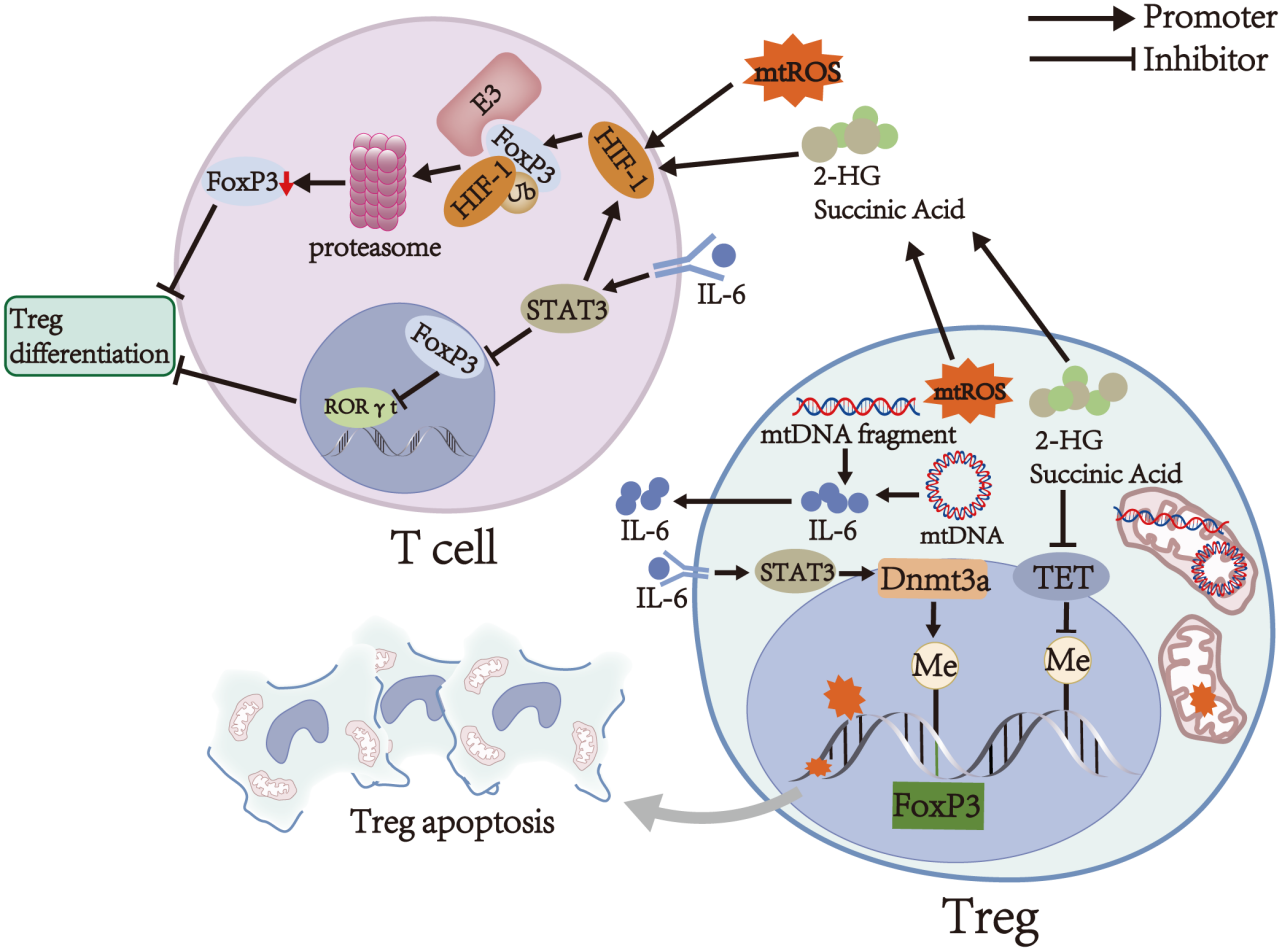
Main mechanisms of Treg regulation by mitochondria. mtROS induces DNA breaks, and the latter can induce Treg apoptosis. Moreover, mtROS upregulates and stabilizes HIF-1, which can downregulate FoxP3 levels and inhibit Treg differentiation by binding to FoxP3 in T cells to degrade the latter via the ubiquitin-proteasome pathway. mtDNA promotes the secretion of IL-6, which activates STAT3 and promotes *Foxp3* locus methylation in Tregs in a Dnmt3a-dependent manner, thereby decreasing the expression of FoxP3 and weakening the immunosuppressive function of Tregs. On the other hand, IL-6 activates STAT3 in T cells to attenuate RORγt inhibition by FoxP3 and promote the transcription of HIF-1, thereby inhibiting Treg differentiation. Succinate and 2-HG inhibit TET, thereby causing DNA hypermethylation in specific regulatory regions of Tregs and impairing the immunosuppressive function of Tregs. Furthermore, succinate and 2-HG stabilize HIF-1 and inhibit Treg differentiation.

mtROS upregulates the transcription of hypoxia-inducible factor 1α (HIF-1α) subunits through NF-κB activation and stabilizes HIF-1α subunits by inhibiting prolyl hydroxylase and asparaginyl hydroxylase (38–40). Stabilized HIF-1α subunits dimerize with HIF-1β subunits (also known as aryl hydrocarbon receptor nuclear translocator) to form HIF-1 (41). Subsequently, HIF-1 translocates into the nucleus and induces the transcription of genes encoding glycolytic enzymes and glucose transporters, leading to a metabolic shift from OXPHOS to glycolysis in the early stages of T cell differentiation (42, 43). Data from several animal studies suggest that glycolysis reduces the expression of FoxP3, CD25, PD-1, CTLA-4, and ICOS (**Table 1**), disrupts their stability, and inhibits induced Treg (iTreg) differentiation, thereby impairing the immunosuppressive function of iTregs and decreasing the number of iTregs (15, 25, 43, 49). Moreover, excessive activation of glycolysis reduces the stability of thymus-derived Tregs (tTregs) by downregulating the expression of FoxP3 and CD25 and converts tTregs into pathogenic cells with effector or memory T cell phenotype (25, 50–52). These cells contribute to autoimmune diseases of the CNS by producing pro-inflammatory cytokines, such as IFN-γ and IL-17 (51, 53–55). However, recent studies have shown that glycolysis is essential for Treg differentiation and function. For example, some *in vitro* studies have shown that glycolysis can induce the differentiation of naive T cells into iTreg and upregulate the expression of CTLA-4, PD-1, CD39, and ICOS (**Table 1**) to maintain the immunosuppressive function of iTregs (24, 56). These processes are achieved by regulating the expression of *FoxP3* exon 2 splicing variants via glycolytic enzyme enolase-1 (24, 56). In addition, glycolysis is required for the optimal expression of the inhibitory molecules CTLA-4 and ICOS and is essential for the immunosuppressive function of tTregs (24, 57). The controversial roles of glycolysis in Treg differentiation and function may be due to differences in the origin of Tregs (thymic or peripheral T cells, human or animal), external and internal environments, metabolic requirements, and cytokines environments. Therefore, the effect of glycolysis on Tregs still needs to be explored. Interestingly, due to the dimerization of HIF-1α subunits and HIF-1β subunits and aryl hydrocarbon receptor degradation by HIF-1α subunits via the ubiquitin-proteasome pathway, the binding rate of aryl hydrocarbon receptor to HIF-1β subunits decreases, causing reduced transcriptional activity of genes encoding ectoenzymes and IL-10 during Treg differentiation and eventually inhibiting Treg differentiation and reducing the number of Tregs (58, 59). Furthermore, HIF-1 binds to the transcription factor FoxP3 in T cell cytoplasm to degrade the latter via the ubiquitin-proteasome pathway, thereby downregulating FoxP3 levels and inhibiting Treg differentiation (**Figure 2**) (60, 61). However, the exact role of HIF-1 in Tregs remains controversial. Clambey et al. (62) and Flück et al. (63) found that HIF-1 can promote the proliferation of Tregs by inducing FoxP3 transcription, thus inhibiting T cell-mediated colitis. The contradictory roles may be due to the tissue heterogeneity of Tregs, suggesting that HIF-1 expression is essential for Tregs in specific tissues. In addition to inhibiting Treg differentiation by stabilizing HIF-1, mtROS can induce DNA breaks. Subsequently, DNA breaks induce Treg apoptosis by initiating a DNA damage response, ultimately decreasing Treg number (**Figure 2**) (31, 64).

**Table 1:** Genes and molecules associated with Treg function.

Gene	Encoding protein	Function	References
CTLA-4	CTLA-4	CTLA-4, the co-inhibitory molecule of Tregs, inhibits the maturation and antigen-presenting ability of dendritic cells by binding to the co-stimulatory molecule CD80/CD86 on the surface of dendritic cells.	(1)
PDCD1	PD-1	PD-1 maintains immune tolerance by regulating the balance between Tregs and effector T cells.	(44)
ENTPD1	CD39	CD39, the ectoenzyme of Tregs, degrades ATP to AMP and then cooperates with CD73 to mediate immunosuppressive adenosine inhibition on effector T cells.	(1, 45)
NT5E	CD73	CD73, the ectoenzyme of Tregs, promotes adenosine binding to adenosine receptor 2A on the surface of effector T cells by degrading AMP to adenosine, thereby inhibiting the function of effector T cells.	(1, 45)
TIGIT	TIGIT	TIGIT^+^ Tregs inhibit the production of IL-12 and IL-23 in dendritic cells by promoting IL-10 and FGL2 secretion, thereby selectively suppressing Th1 and Th17 cell responses.	(46, 47)
FGL2	FGL2	FGL2 inhibits B cell proliferation and differentiation and plasma cell apoptosis, and inhibits maturation and antigen presentation of dendritic cells by binding to the FcγRIIb receptor, thereby exerting the immunosuppressive activity of Tregs.	(48)

### 2.2 Effect of mtDNA release on Tregs

During mitochondrial dysfunction, mtDNA is cleaved into small fragments by endonucleases. The fragments are released into the cytosol through the permeability transition pore complex (PTPC, including mitochondrial permeability transition pores/MPTP and voltage-dependent anion channels/VDAC) (**Figure 1**) (65). Moreover, BAX and BAK oligomerize in the outer mitochondrial membrane and form BAX/BAK pores, which allow the inner mitochondrial membrane to herniate into the cytosol and release mtDNA (**Figure 1**) (66). Cytosolic mtDNA activates the inflammasome NLRP3, which in turn increases the release of mtDNA through a positive feedback mechanism (65, 67, 68). In the EAE model, the increase of mtDNA fluorescent particles in the cytoplasm confirmed the release of mtDNA fragments (31).

As an upstream effector, cytosolic mtDNA promotes the secretion of IL-6 and IL-10 by activating several signal pathways, including cGAS-STING and TLR9-MyD88 signals, finally affecting the number and function of Tregs (**Figure 1**) (68–70). Among them, IL-10 is an immunosuppressive cytokine that suppresses the activation of autoreactive T cells and downregulates pro-inflammatory cytokines to enhance the immunosuppressive function of Tregs in stress conditions (1, 71). In contrast, IL-6 activates STAT3 and promotes *Foxp3* locus methylation in Tregs in a DNA methyltransferase 3a (Dnmt3a)-dependent manner, thereby decreasing the expression of FoxP3 (**Figure 2**) (55, 72). Low FoxP3 expression can downregulate co-inhibitory molecules and ectoenzymes on the surfaces of Tregs and turn Tregs into pathogenic cells with effector or memory T cell phenotype (20, 53, 55). On the other hand, IL-6 activates STAT3 in T cells by binding to IL-6 receptor and gp130, which can attenuate RORγt inhibition by FoxP3 and promote the transcription of HIF-1α subunits (**Figure 2**) (21, 60, 73). These alterations finally inhibit Treg differentiation and decrease Treg number (43, 61). Thus, the influence of mtDNA on the number and function of Tregs may depend on the balance between the mechanisms mentioned above.

### 2.3 Impairment of mitochondrial metabolic pathways decreases the number of Tregs and impairs their function

Recent studies have shown that Tregs mainly rely on high levels of FAO and modest glycolysis to meet their energy requirements in steady-state conditions (15, 16). FAO is also necessary for T cell differentiation to Tregs (74, 75). During FAO-driven OXPHOS, fatty acids increase the stability and immunosuppressive activity of Tregs by upregulating FoxP3 transcription and inducing CD25 and STAT5 expression (23, 76). At the same time, FoxP3 increases the transcription of FAO and OXPHOS-related genes and inhibits glycolysis by binding to the *Myc* promoter and downregulating the expression of Myc in Tregs, establishing a positive feedback loop to maximize the immunosuppressive function of Tregs (22, 25, 77). Carnitine palmitoyltransferase 1 (CPT1) on the outer mitochondrial membrane is a rate-limiting enzyme in FAO (**Figure 1**). Previous studies have demonstrated that CPT1 inhibitors can prevent Treg differentiation and significantly reduce the expression of granzymes, ectoenzymes, and co-inhibitory molecules in Tregs by suppressing FAO, thereby reducing the number and immunosuppressive activity of Tregs (15, 78, 79). Therefore, FAO impairment due to reduced or defective CPT1 activity reduces the number and immunosuppressive activity of Tregs. In addition, fatty acid concentrations in patients with MS are lower than those in healthy individuals, thereby decreasing FAO in Tregs and weakening the inhibitory function of Tregs (23).

OXPHOS is a critical metabolic pathway for the differentiation and immunosuppressive function of Tregs (15–17). Therefore, decreased activity and expression of respiratory chain complexes in autoimmune diseases of the CNS, such as MS and NMOSD, can contribute to the abnormal number and function of Tregs (32, 33, 77, 80, 81). Weinberg et al. (81) found that accumulation of succinate and 2-hydroxyglutarate (2-HG) can inhibit TET by competing with α-ketoglutarate in respiratory chain complex III-deficient Tregs (**Figure 1**), thereby causing DNA hypermethylation in specific regulatory regions of Tregs and reducing the expression of *PDCD1* (encoding PD-1), *NT5E* (encoding CD73), *TIGIT*, and *FGL2* genes associated with the immunosuppressive function of Tregs (**Figure 2**) (82, 83). These alterations finally downregulate co-inhibitory molecules and ectoenzymes, such as PD-1, CD73, TIGIT, and FGL2, and impair the immunosuppressive function of Tregs (**Table 1**) (44–46, 48, 81). On the other hand, succinate and 2-HG stabilize HIF-1α subunits by inhibiting prolyl hydroxylases, thereby inhibiting Treg differentiation and decreasing Treg number (**Figure 2**) (83–85). Similar to respiratory chain complex III inhibition, respiratory chain complex I inhibition can downregulate the expression of FoxP3 and decrease the number of Tregs (16). In addition, Angelin et al. (77) found that Tregs with mitochondrial ND6 gene mutations have reduced immunosuppressive function due to the inability of respiratory chain complex I to oxidize NADH to NAD.

### 2.4 Impairment of mitophagy decreases the number of Tregs

Mitophagy, a specific form of autophagy, is essential for clearing damaged mitochondria and maintaining cell homeostasis (86). Mitochondrial depolarization induces the ubiquitination of mitochondrial outer membrane proteins and promotes the recruitment of mitophagy receptors, followed by autophagosome formation to degrade damaged mitochondria (86). Therefore, impaired initiation of mitophagy, incorrect autophagosome formation, or aberrant lysosomal degradation in autoimmune diseases of the CNS can impair mitophagy in Tregs (**Figure 1**) (31, 87).

Crosstalk between mitochondria and lysosomes has been demonstrated. Lysosomal dysfunction induces mitochondrial defect and vice versa (88, 89). Mitochondrial dysfunction in Tregs of EAE mice can decrease the activity of several hydrolases in lysosomes and downregulate the expression of Rab7, which regulates the fusion of autophagosomes with lysosomes, thus preventing lysosomal degradation of damaged mitochondria (31, 88, 90). In addition, downregulation of the AMPK-PIKFYVE-PtdIns (3, 5) P2-MCOLN1 pathway in the presence of damaged respiratory chain complexes leads to lysosomal calcium accumulation and impairs lysosomal hydrolysis, thereby impairing mitophagy (91, 92). In addition to lysosomal dysfunction, significantly reduced expression of autophagy protein LC3-II in Tregs in patients with myasthenia gravis can impair autophagosome formation and reduce the number of autophagosomes, eventually impairing mitophagy (87). Thanks to the damaged mitophagy of Tregs, the accumulation of damaged mitochondria increases mtROS production and exacerbates mitochondrial oxidative stress, thereby forming a vicious circle (31). Eventually, mtROS accumulation induces DNA breaks, and DNA damage induces Treg apoptosis by initiating a DNA damage response (31, 64).

## 3 Mitochondrial-regulated Tregs may be potential therapeutic targets for autoimmune diseases of the CNS

Accumulation of mtROS decreases the number of Tregs; thus, a better understanding of the role of mitochondria-specific antioxidants in reducing mtROS may provide a new modality for immunotherapy of autoimmune diseases of the CNS. Animal studies applying mitochondrial antioxidants have reported promising results. Mito-TEMPO, a mitochondria-targeting antioxidant mimicking superoxide dismutase, can restore lysosome function and inhibit Treg apoptosis in the EAE mice model by reducing mtROS levels and mitigating mtROS damage, thereby inhibiting effector T cell infiltration into the spinal cord and increasing Treg infiltration to alleviate the symptoms (**Table 2**) (31, 93). The ability of superoxide dismutase mimics to delay the progression of EAE suggests that these novel antioxidants can be applied to autoimmune diseases of the CNS, such as NMOSD and autoimmune encephalitis, in the future. Furthermore, Cyclosporin A (CsA), an MPTP inhibitor, can reduce the production of mtROS and attenuate mitochondrial dysfunction by inhibiting MPTP opening through the blockade of the interaction of cyclophilin D with adenine nucleotide translocator (**Table 2**) (94, 101). Studies have found that CsA can induce Treg proliferation and prevent T cell proliferation by inhibiting calcineurin, thus ameliorating the symptoms of EAE, MS, and NMOSD (95, 96, 102). However, some immunosuppressive effects of CsA may be caused by MPTP inhibition, but this possibility has not been investigated and remains to be explored in the future. In addition, genetic or pharmacological downregulation of mtROS lowers HIF-1α levels, and HIF-1α-deficient mice with an increased number of Tregs are resistant to EAE (61, 103, 104). However, these results do not demonstrate a direct link between reduced mtROS levels, lowered HIF-1α levels, and an increased number of Tregs. In the future, it is necessary to explore whether mitochondrial antioxidants inhibit HIF-1α subunits and whether this inhibition affects Tregs in autoimmune diseases of the CNS.

**Table 2:** Experimental therapeutic drugs targeting mitochondria of Tregs for autoimmune diseases of the CNS.

Drug	Target	Mechanisms	References
Mito-TEMPO	Mitochondria	Reduce mtROS levels and mitigate mtROS damage	(31, 93)
Cyclosporin A	MPTP and calcineurin	Inhibit MPTP opening and calcineurin	(94–96)
VBIT-4*	VDAC1	Prevent VDAC1 oligomerization	(97)
VBIT-12*	VDAC1	Prevent VDAC1 oligomerization	(98)
2-deoxyglucose	Hexokinase	Inhibit glycolysis	(43)
Rapamycin	mTOR	Inhibit glycolysis and enhance OXPHOS	(43, 49)
Dichloroacetate	Pyruvate dehydrogenase kinase	Inhibit glycolysis and enhance OXPHOS	(16, 99)
IL-15*	mitochondrial transcription factor A, and peroxisome proliferator-activated receptor-γ coactivator-1α	Improve mitochondrial mass and OXPHOS	(100)

“*” means there is no evidence in preclinical models of autoimmune diseases in the CNS, and future studies are needed to explore the pharmacologic effects of these drugs in autoimmune diseases of the CNS.

mtDNA release affects the number and function of Tregs and induces the inflammatory response in autoimmune diseases of the CNS. Therefore, targeting mtDNA release may be another novel therapeutic approach for treating autoimmune diseases of CNS. VBIT-4 and VBIT-12, two VDAC1 oligomerization inhibitors, prevent VDAC1 oligomerization by directly interacting with VDAC1, thereby inhibiting apoptosis, reducing mtDNA release, inhibiting inflammatory cell infiltration and inflammasome NLRP3 activation, protecting against mitochondrial dysfunction, and reducing inflammatory response and disease severity (**Table 2**) (97, 98). This VDAC1-based treatment strategy has been effective in some animal models of autoimmune diseases, such as inflammatory bowel disease, systemic lupus erythematosus, and type 2 diabetes (97, 98, 105). Future studies on autoimmune diseases of the CNS are needed to determine the efficacy of VDAC1-based treatment for these diseases.

Increased mitochondrial metabolism promotes Treg differentiation, while inhibition of mitochondrial metabolism or increased glycolysis inhibits Treg differentiation. Therefore, shifting cellular energy metabolism to OXPHOS may be a treatment strategy for autoimmune diseases of the CNS. Animal studies of cellular metabolic reprogramming have yielded promising results in autoimmune diseases of the CNS. For example, inhibition of glycolysis by HIF-1α gene knockout or application of 2-deoxyglucose or rapamycin can promote Treg differentiation, increase the number of Tregs, and reduce spinal cord inflammation in EAE mice (**Table 2**) (43, 49). Similarly, decreasing pyruvate dehydrogenase kinase activity genetically (gene knockout) or pharmacologically (by dichloroacetate) can enhance OXPHOS levels, promote Treg differentiation, increase the number of Tregs, and protect mice against EAE (**Table 2**) (16, 99). The development of this therapeutic strategy requires further research. In the future, we should explore whether these drugs affecting cellular metabolic reprogramming can modulate the activity of Tregs in autoimmune diseases of the human CNS. Furthermore, IL-15 has been shown to improve mitochondrial mass and OXPHOS in Tregs from HIV-infected immune non-responders by inducing the expression of mitochondrial transcription factor A and peroxisome proliferator-activated receptor-γ coactivator-1α (**Table 2**) (100). Currently, IL-15 and some of its derivatives, such as IL-15 super-agonists, are in clinical trials for cancer and AIDS. Future studies are needed to investigate the immunologic role of IL-15 in autoimmune diseases of the CNS.

## 4 Conclusion and prospect

Recent studies have investigated the role of mitochondrial regulation on Treg number and function. However, the details of the mitochondrial regulation process remain to be elucidated. For example, the type of E3 ligase responsible for HIF-1-mediated ubiquitination of FoxP3 is still unclear. Similarly, the effects of glycolysis on Tregs, the mechanisms by which mtDNA affects Tregs and the mechanisms by which respiratory chain complex I damage causes Treg dysfunction need more studies. Moreover, whether drugs targeting mitochondria can improve human autoimmune diseases of the CNS by selectively modulating Treg activity remains to be explored.

In conclusion, understanding the mechanisms by which mitochondrial dysfunction affects the number and function of Tregs in autoimmune diseases may pave the way for developing new therapeutic approaches. Future in-depth studies in this field will be a significant entry point for exploring the molecular mechanisms and therapeutic targets in autoimmune diseases of the CNS.

## Author contributions

AH: Writing – original draft, Writing – review & editing. TP: Writing – review & editing. YYX: Writing – review & editing. WZ: Writing – review & editing. WS: Writing – review & editing. YX: Writing – review & editing. YM: Writing – review & editing. CW: Writing – review & editing. NX: Writing – review & editing.
